# The Association between Serum Albumin and Post-Operative Outcomes among Patients Undergoing Common Surgical Procedures: An Analysis of a Multi-Specialty Surgical Cohort from the National Surgical Quality Improvement Program (NSQIP)

**DOI:** 10.3390/jcm11216543

**Published:** 2022-11-04

**Authors:** Cole A. Nipper, Kelvin Lim, Carlos Riveros, Enshuo Hsu, Sanjana Ranganathan, Jiaqiong Xu, Michael Brooks, Nestor Esnaola, Zachary Klaassen, Angela Jerath, Amanda Arrington, Christopher J. D. Wallis, Raj Satkunasivam

**Affiliations:** 1 Department of Urology, Houston Methodist Hospital, Houston, TX 77030, USA; 2 Center for Health Data Science and Analytics, Houston Methodist Hospital, Houston, TX 77030, USA; 3 Department of Surgical Oncology, Houston Methodist Hospital, Houston, TX 77030, USA; 4 Division of Urology, Medical College of Georgia, Augusta University, Augusta, GA 30912, USA; 5 Department of Anesthesia, Sunnybrook Health Sciences Center, Toronto, ON M4N 3M5, Canada; 6 Division of Urology and Surgical Oncology, Department of Surgery, Princess Margaret Cancer Centre, University Health Network, University of Toronto, Toronto, ON M5G 2C4, Canada; 7 Division of Urology, University of Toronto, Toronto, ON M5G 1X6, Canada; 8 Division of Urology, Mount Sinai Hospital, Toronto, ON M5G 1X5, Canada

**Keywords:** serum albumin, preoperative risk stratification, surgical outcomes, NSQIP, albumin

## Abstract

While studies have demonstrated an association between preoperative hypoalbuminemia and adverse clinical outcomes, the optimal serum albumin threshold for risk-stratification in the broader surgical population remains poorly defined. We sought define the optimal threshold of preoperative serum albumin concentration for risk-stratification of adverse post-operative outcomes. Using the American College of Surgeons National Surgical Quality Improvement Program Database, we identified 842,672 patients that had undergone a common surgical procedure in one of eight surgical specialties. An optimal serum albumin concentration threshold for risk-stratification was determined using receiver-operating characteristic analysis. Multivariable logistic regression analysis was used to evaluate the odds of adverse surgical events; a priori defined subgroup analyses were performed. A serum albumin threshold of 3.4 g/dL optimally predicted adverse surgical outcomes in the broader cohort. After multivariable analysis, patients with hypoalbuminemia had increased odds of death within 30 days of surgery (odds ratio [OR] 2.01, 95% confidence interval [CI] 1.94–2.08). Hypoalbuminemia was associated with greater odds of primary adverse events among patients with disseminated cancer (OR 2.03, 95% CI 1.88–2.20) compared to patients without disseminated cancer (OR 1.47, 95% CI 1.44–1.51). The standard clinical threshold for hypoalbuminemia is the optimal threshold for preoperative risk assessment.

## 1. Introduction

Serum albumin is a negative acute-phase protein and its concentration is generally reduced during hyperinflammatory states. Like many laboratory parameters, the historical definition of hypoalbuminemia is derived empirically from the distribution of serum albumin concentration in a representative population [1]. The clinical implication of pathologic hypoalbuminemia both as a prognosticator and as an independent measure of disease progression has been studied extensively. Hypoalbuminemia is associated with increased mortality among patients with chronic disease and among critically ill hospitalized patients [2,3,4,5,6,7,8,9,10,11,12,13]. An association between preoperative hypoalbuminemia and adverse surgical outcomes has been also observed [14,15,16,17]. The persistent association between adverse clinical outcomes and hypoalbuminemia in a variety of clinical contexts suggest that the preoperative assessment of serum albumin concentration may be a robust and clinically useful predictor of adverse surgical outcomes.

In a large prospective observational study, researchers enrolled 54,215 patients undergoing noncardiac surgery in Veterans Affairs (VA) medical centers and determined that, after controlling for patient-specific clinical factors, pre-operative serum albumin was the single strongest predictor of morbidity and mortality [18]. They also found that preoperative serum albumin was most predictive of systemic sepsis, acute renal failure, coma, and failure to wean from ventilation. While this study population was sufficiently large, the generalizability of these data is limited given the predominant VA cohort as well as due to the exclusion of cardiac surgery patients. Although it has been shown that serum albumin concentration may be a poor predictor of frank nutritional status among patients with chronic inflammation or serious illness [19], multiple studies have observed an association between preoperative hypoalbuminemia and adverse outcomes in patients undergoing oncological surgery [20,21].

To address shortcomings in prior literature and to explore the clinical utility of preoperative serum albumin among patients at a population level, we evaluated the association between hypoalbuminemia in the 30-day preoperative period and the occurrence of adverse surgical outcomes among a large multi-disciplinary cohort generated from the American College of Surgeons (ACS) National Surgical Quality Improvement Program (NSQIP) database. We sought to define the clinically significant laboratory value of albumin predictive of adverse post-operative outcomes and examined the heterogeneity of the association between preoperative hypoalbuminemia and adverse outcomes by *a priori* defined subgroups based on surgical specialties or disseminated cancer status.

## 2. Materials and Methods

### 2.1. Study Subjects

The study cohort was generated using the ACS NSQIP participant use files from 2005–2018. Patients 18 years or older were included in the cohort if they underwent cardiac surgery, general surgery, gynecological surgery, neurosurgery, orthopedic surgery, thoracic surgery, urological surgery, or vascular surgery. The specific CPT codes used to generate the multi-surgical cohort are listed in Table S1. Patients were excluded from the study if they were missing any of the outcome data for the 30-day postoperative period. They were also excluded if they were missing any of the covariates of interest (age, gender, race, American Society of Anesthesiologists [ASA] status, smoking status, diabetes treatment, height, and weight) or primary data corresponding to pre-operative serum albumin level. To account for its half-life, patients with preoperative serum albumin labs drawn greater than 30 days prior to surgery were also excluded.

### 2.2. Outcomes

Primary outcomes included major postoperative adverse events such as death, cardiac events (myocardial infarction, cardiac arrest requiring CPR), neurological events (stroke, cerebrovascular accident [CVA]), and unplanned reoperation within 30 days. Secondary outcomes included postoperative complications such as surgical site infections (SSI; superficial, deep, and organ space SSI, as well as wound disruption), pneumonia, urinary tract infection, septic events (sepsis, septic shock), pulmonary complications (unplanned intubation, mechanical ventilation > 48 h), venous thromboembolism (pulmonary embolism, deep venous thromboembolism [DVT] requiring therapy), acute renal failure, bleeding necessitating transfusion, unplanned readmission, and prolonged length of stay (pLOS). Because length of stay is procedure dependent, pLOS was determined by operationalizing length of stay among the individual CPT codes used for cohort creation and defined as a length of stay above the 75th percentile. Due to insufficient granularity, a small proportion of patients that underwent surgeries corresponding to CPT codes that had fewer than ten patients were excluded from the pLOS analysis (*n* = 1815). A composite primary adverse event is reported and is defined as ‘positive’ if any primary adverse event occurs. An analogous composite secondary adverse event is also reported. Individual primary and secondary outcomes are reported for completeness.

### 2.3. Exposure

The primary exposure was preoperative serum albumin concentration, measured within 30 days of the date of surgery. Per ACS-NSQIP guidelines, only the most recent preoperative serum albumin concentration value is reported.

### 2.4. Covariates

Multivariable models were adjusted according to the standard demographic and clinical covariates available for all patients including: age (0–39, 40–49, 50–59, 60–69, 70–79, 80+), race, sex, body mass index (BMI: <18.5, 18.5–24.9, 25–29.9, 30+) functional health status (independent versus partially or totally dependent), ASA classification, current smoking status (active smoker within one year), bleeding disorder, diabetes mellitus (requiring oral agent or insulin), hypertension requiring medication, ventilator dependence, currently requiring or on dialysis, severe chronic obstructive pulmonary disease (COPD), congestive heart failure (within 30 days prior to surgery), ascites (within 30 days prior to surgery), acute renal failure (preoperative), sepsis within 48 h prior to surgery, preoperative transfusion (≥1 unit of whole/packed red blood cells within 72 h prior to surgery), immunosuppressive therapy (steroid or immunosuppressant use for a chronic condition), total operation time, surgical specialty, and presence of disseminated cancer. Global multivariable models were adjusted on all covariates. Multivariable subgroup analysis models were adjusted on all covariates except the subgroup of interest.

### 2.5. Statistical Analysis

In order to determine the most optimal threshold to differentiate patients at high-risk and low-risk for post-operative adverse events on the basis of preoperative serum albumin concentration, we generated 100 multivariable logistic regression models for each outcome, treating serum albumin as a binary classifier that was incremented in consecutive steps of 0.1 g/dL, starting at 0. Each model was adjusted on all covariates. The performance of the models was evaluated by calculating the area under the receiver operating characteristic (ROC) curve, which was estimated using the trapezoid rule. An ‘optimal’ value defined as “the threshold that maximized the area under the ROC curve” was calculated for each outcome. This same analysis was performed in a subgroup containing only patients with disseminated cancer.

Using the optimal threshold that best risk-stratified patients on the basis of composite adverse events, patients were dichotomized into high-risk and low-risk groups. Descriptive statistics were used to evaluate differences in demographic and clinical characteristics among the two groups. Categorical variables were reported in frequency and proportion, while continuous variables were reported in median and interquartile range. Categorical variables were compared using Pearson’s chi-square test (or Fisher’s exact test where appropriate), and continuous variables were compared using the Wilcoxon signed-rank test. Similarly, crude rates of adverse surgical outcomes were compared between the two groups. We also compared crude rates of adverse surgical outcomes in a subgroup of patients with and without disseminated cancer.

Using the optimal threshold as a binary classifier, multivariable logistic regression analysis was used to evaluate the risk stratification capacity of the optimal threshold by calculating the odds of 30-day adverse surgical outcomes after adjusting for a priori selected covariates. Subgroup analyses were performed to evaluate the risk stratification capacity of the optimal threshold among patients with and without disseminated cancer, as well as among various surgical specialties. Odds ratios (OR) and 95% confidence intervals (CI) are reported for each outcome in the multivariable logistic regression models. All reported p-values were two-sided with significance below 0.05.

To evaluate the heterogeneity of association between serum albumin concentration and adverse surgical events, albumin was included as a restricted linear spline term with 6 knots in a logistic regression analysis. Knots were placed at 1.4, 2.4, 3.4, 4.4, 5.4, and 6.4 g/dL, respectively. The linear segment coefficients are reported as ORs with their respective 95% CIs.

In all multivariable models, observations with missing outcomes or co-variates were dropped. Multicollinearity was handled by the statistical software by excluding co-variates that caused collinearity. All analyses maintained the standard definition of statistical significance as a 2-tailed α risk of 0.05 or less. Statistical analyses were performed with STATA 17 (StataCorp. 2021. Stata Statistical Software: Release 17. College Station, TX, USA: StataCorp LLC).

## 3. Results

### 3.1. Receiver Operating Characteristic Analysis

**Figure 1:**
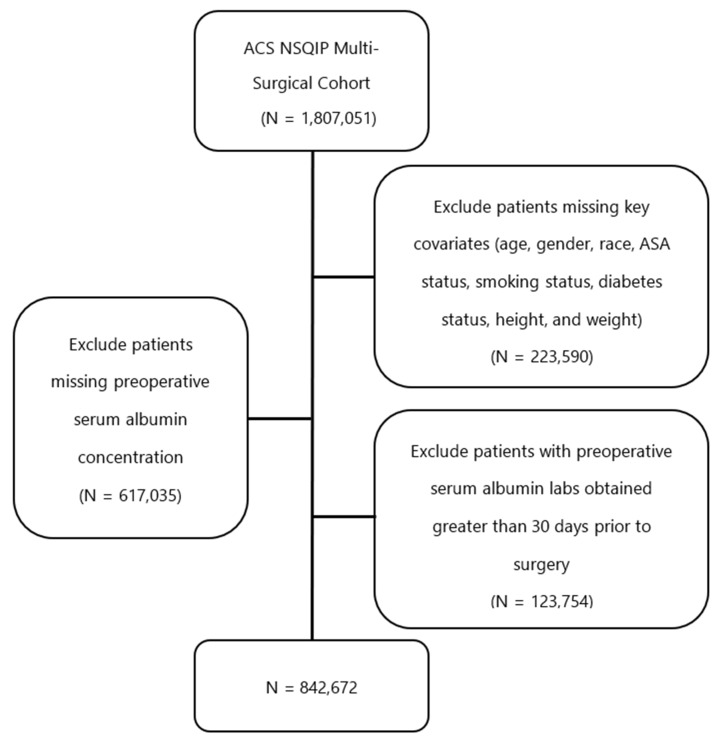
Cohort Creation Algorithm—A total of 842,672 patients were included in the study after excluding patients missing key covariates or missing serum albumin concentration within 30 days of surgery. ACS–American College of Surgeons; NSQIP—National Surgical Quality Improvement Program; ASA—American Society of Anesthesiologists.

**Figure 2:**
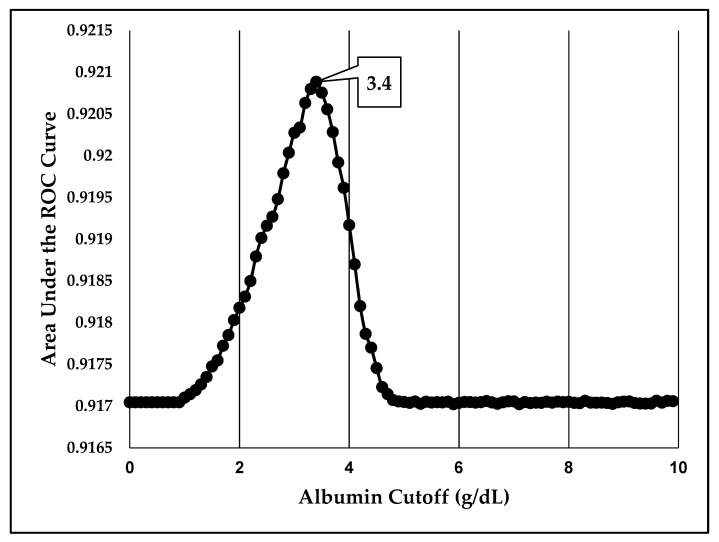
Optimization of preoperative serum albumin cutoff to predict death in the 30-day postoperative period—A preoperative serum albumin concentration threshold of 3.4 g/dL most optimally predicts death after multivariable adjustment. ROC—receiver operating characteristic.

We identified 842,672 patients in the ACS-NSQIP database who had undergone surgical procedures corresponding to one of eight surgical specialties and had serum albumin labs drawn no more than 30 days prior to the surgery (Figure 1). After ROC curve analysis, we identified the preoperative serum albumin concentration cutoff that was most predictive of every adverse surgical event (Table 1). A preoperative serum albumin concentration cutoff of 3.4 g/dL most optimally predicted death in the immediate 30-day postoperative period (Figure 2). We observed other important trends in this analysis: 13 out of 21 outcomes were optimally predicted by a preoperative serum albumin cutoff of 3.4 g/dL +/− 0.1, including death, pLOS, and 30-day unplanned reoperation. Furthermore, both composite primary and secondary adverse events were similarly predicted by a cutoff of 3.4 g/dL +/− 0.1 (3.5 and 3.3 g/dL respectively). Only four out of 21 outcomes were optimally predicted by a serum albumin cutoff of less than 3.4 g/dL. The remainder cutoffs were within the normal range of serum albumin concentration. We therefore defined the optimal cutoff as the standard threshold for hypoalbuminemia, 3.4 g/dL. In a subgroup analysis, we found that the optimal serum albumin cutoffs in patients with disseminated cancer was lower in four out of five primary adverse events. A cutoff of 3.0 g/dL was most predictive of composite primary adverse events in this subgroup. (Table S2).

### 3.2. Descriptive Analysis of Cohort

Of the 842,672 patients in the ACS-NSQIP cohort, 148,478 (17.6%) had high-risk preoperative serum albumin concentration (≤3.4 g/dL; hereinafter referred to as hypoalbuminemia) and 694,194 (82.4%) had low risk preoperative serum albumin concentration. Patients with hypoalbuminemia were more likely to be older, non-white, active smokers, and male (Table 2). Furthermore, patients with hypoalbuminemia had significantly lower BMI, worse functional health status, and elevated ASA classification in univariate comparisons. Among general surgery or vascular surgery subgroups, there was an increased (65.2% and 21.7%, respectively) proportion of patients with hypoalbuminemia, whereas in the orthopedic surgery subgroup, there was a decreased (5%) proportion of patients with hypoalbuminemia.

### 3.3. Univariate Analysis of Adverse Surgical Outcomes

**Figure 3:**
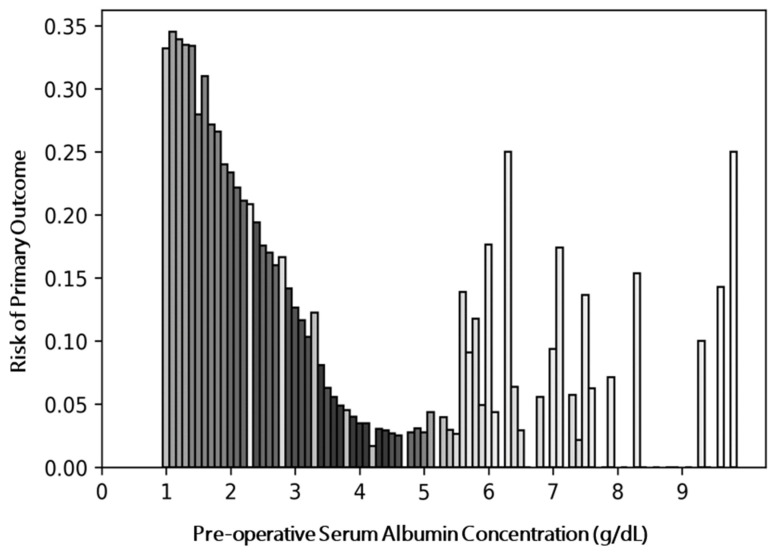
Unadjusted Risk of Composite Primary Outcomes as a Function of Preoperative Serum Albumin—The composite risk of primary outcomes was calculated after binning the cohort according to serum albumin concentration in steps of 0.1 g/dL. Bar shading corresponds to the number of patients in each bin, with darker shades indicating more patients and lighter shades indicating fewer patients.

We identified a greater composite risk of primary adverse events among patients with hypoalbuminemia. We found that this composite risk decreases as preoperative serum albumin is increased in the high-risk serum albumin range (Figure 3). Patients with hypoalbuminemia had an increased relative risk of all outcome measures considered both individually and in composite (Figure S1, Table 3). Among patients with disseminated cancer, patients with hypoalbuminemia demonstrated an increased proportion of all adverse outcomes except unplanned 30-day readmission (Table S3).

### 3.4. Multivariable Analysis of Adverse Surgical Outcomes

After multivariable adjustment for relevant demographic and clinical factors, we found that patients with hypoalbuminemia had significantly greater odds of experiencing primary adverse surgical events, including death (OR 2.01, 95% CI 1.94–2.08), cardiac arrest requiring CPR (OR 1.46, 95% CI 1.38–1.55), stroke/CVA (OR 1.36, 95% CI 1.24–1.48), and reoperation (OR 1.31, 95% CI 1.27–1.36), with the exception of myocardial infarction (Figure S2, Table S4). Furthermore, we found that patients with hypoalbuminemia were also found to have significantly greater odds of experiencing secondary adverse surgical events (Figure S3, Table S4).

### 3.5. Adverse Surgical Outcomes Stratified by Surgical Specialties

We found marginal heterogeneity in the association between hypoalbuminemia and adverse surgical events across the different surgical sub-specialties (Figure S4). Independent of surgical specialty, patients with hypoalbuminemia had significantly greater odds of death and composite secondary adverse events, as well as increased odds of experiencing septic shock, transfusions, and pLOS (Table 4). In all specialties except neurosurgery, patients with hypoalbuminemia had greater odds of developing pneumonia or ventilator dependence for greater than 48 h. Patients with hypoalbuminemia also had greater odds of developing sepsis or acute renal failure in all specialties except neurosurgery and urology.

### 3.6. Adverse Surgical Outcomes Stratified by Disseminated Cancer

In this cancer-specific analysis, we observed that hypoalbuminemia was associated with increased odds of the primary composite adverse surgical events among patients with both disseminated cancer (OR 2.03, 95% CI 1.88–2.20) and without disseminated cancer (OR 1.47, 95% CI 1.44–1.51). This finding was seen across all adverse events except myocardial infarction. Overall, hypoalbuminemia was associated with significantly greater odds of adverse surgical outcomes in the disseminated cancer group in several outcome measures including total odds of a secondary adverse event (OR 2.63, 95% CI 2.48–2.78) and death (OR 3.14, 95% CI 2.82–3.50) (Table S5).

### 3.7. Spline Analysis Evaluating Heterogeneity in the Association between Serum Albumin and Odds of Adverse Events

In this spline analysis, we observed that an increase in preoperative serum albumin concentration by 1 g/dL was associated with decreased odds of death among patients with preoperative hypoalbuminemia (≤3.4 g/dL) (Table S6), even after controlling for relevant clinical covariates. Moreover, a similar increase in serum albumin among patients with serum albumin concentration between 3.4 and 4.4 g/dL was associated with a 55% lower odds of death. In patients with serum albumin concentration > 4.4 g/dL, further increases in serum albumin were not found to be associated with decreased odds of death.

With the exception of patients with serum albumin < 1.4 g/dL or >6.4 g/dL, we observed this same trend among composite primary adverse events, with the nadir of the spline curve in the 3.4–4.4 g/dL range. In the spline curve corresponding to composite secondary adverse events, the nadir was observed in the 2.4–3.4 g/dL albumin range.

## 4. Discussion

We sought to identify an optimal preoperative serum albumin threshold for surgical risk stratification. We found that a threshold of 3.4 g/dL +/− 0.1 g/dL most optimally distinguishes high-risk and low-risk surgical patients in the general population, while a lower threshold of 3.0 g/dL was more predictive of primary adverse surgical events among patients with disseminated cancer. We therefore concluded that the standard threshold for hypoalbuminemia is an optimal cut-off for distinguishing high-risk and low-risk surgical candidates.

Being both a common and inexpensive laboratory test, serum albumin is an attractive pre-surgical prognosticator and has been repeatedly shown to correlate with adverse surgical outcomes in both retrospective and prospective studies [22,23]. Our study largely corroborates prior work. In evaluating the utility of hypoalbuminemia as a risk stratification threshold, we found that patients with preoperative hypoalbuminemia had increased rates of adverse surgical outcomes in the immediate 30-day postoperative period, with little heterogeneity across eight different surgical specialties after controlling for clinically relevant covariates, reinforcing many of the findings of Gibbs et al. [18]. We also showed that while a greater proportion of patients with disseminated cancer experienced preoperative hypoalbuminemia, this was independently associated with a significantly greater increase in odds of adverse surgical outcomes including death.

In examining the relationship between preoperative serum albumin concentration and odds of 30-day adverse surgical outcomes, we observed natural inflections in the crude rates of adverse events corresponding to the lower and upper bounds of normal serum albumin concentration. On further analysis, we demonstrated that while increases in serum albumin concentration are generally associated with decreased rates of adverse surgical events among patients with hypoalbuminemia and normal serum albumin, the effect is more pronounced among patients with serum albumin concentration near the low end of normal range (3.4–4.4 g/dL) and is not consistently observed among patients with serum albumin concentration in the high normal range (>4.4 g/dL). A similar finding has been reported previously among a cohort of outpatient surgery patients derived from NSQIP [24]. This trend in the spline curve was observed among composite primary outcomes and death even after controlling for covariates that are highly associated with alterations in albumin concentration such as age and disseminated cancer as well as controlling for factors that directly alter odds of adverse events such as surgical specialty. We therefore hypothesize that patients with serum albumin concentration < 3.4 g/dL may harbor clinical comorbidities (either known or unknown) that place them at increased odds for adverse surgical events. That being said, due to the nature of this study design, we cannot rule out the possibility that increases in albumin concentration may provide a direct protective effect.

Several protective effects of serum albumin have been proposed to explain the persistent association of hypoalbuminemia and worse clinical outcomes after controlling for major clinical covariates [25]. Studies have, however, failed to show a consistent benefit in the routine correction of hypoalbuminemia in critically ill patients outside of specific circumstances [26,27,28]. Additional insight into this phenomenon may be garnered by evaluating the association between change in serum albumin concentration (defined as the delta between preoperative concentration and post-operative concentration) and post-operative adverse events or by evaluating other acute phase proteins. In one prospective trial examining a cohort of 70 patients undergoing abdominal procedures, researchers observed a significant association between post-operative serum albumin concentration drop and odds of post-operative adverse events [29]. Although studies have been performed to evaluate the clinical utility of other acute phase proteins with decreased half-life such as transferrin, retinol-binding protein, and anti-thrombin III, these studies are limited in scope and statistical power. Even though these proteins are not routinely measured preoperatively, an analogous study evaluating the utility of these proteins for preoperative risk stratification may be informative.

The current study is limited by the retrospective nature of its design, and it does not probe any causative or direct protective explanations for the observed correlations. Furthermore, in the multivariable model used to optimize the prognostic threshold of serum albumin concentration, serum albumin appeared to contribute only modestly to the overall accuracy of the model, as evidenced by a relatively limited increase in area under the ROC curve in the optimization analysis. However, this study leverages a large multiprocedural cohort to evaluate the association of serum albumin and adverse surgical outcomes in a variety of clinical contexts, strengthening the clinical applicability of the conclusions. We also sought to extend the clinical utility of the findings by determining the most optimal serum albumin concentration threshold for distinguishing high and low-risk surgical patients based on serum albumin. To our knowledge, this retrospective analysis of preoperative serum albumin is one of the largest and most inclusive of its kind.

The routine use of serum albumin concentration for preoperative risk stratification is controversial not only because serum albumin concentration is affected by numerous physiologic processes, but because the underlying surgical indication should be considered a possible or likely cause of changes in serum albumin concentration [30]. The current work suggests that it is reasonable to consider isolated hypoalbuminemia as a potential indicator of surgical risk which warrants further workup to assess for actionable clinical comorbidities such as malnutrition, liver or kidney disease, and infection. Several findings support this conclusion including the minimal heterogeneity between the association of preoperative serum albumin concentration and adverse outcomes among various specialties as well as the preservation of this association among patients with disseminated cancer, a condition associated with inflammatory and metabolic alterations. There have been few large-scale studies that attempt to identify a firm threshold for risk stratification on the basis of serum albumin concentration. Our work has contributed to this effort by demonstrating that the standard clinical threshold for hypoalbuminemia is the most optimal threshold for fulfilling this role in the broader surgical population.

## 5. Conclusions

In this large population-based study, we demonstrate that a threshold of 3.4 g/dL most optimally distinguishes high and low-risk patients based on serum albumin. We found that that hypoalbuminemia is independently associated with increased odds of 30-day postoperative morbidity and mortality. We showed that increases in serum albumin concentration are associated with decreased odds of adverse surgical events, especially in patients with normal serum albumin concentration. Finally, we showed that hypoalbuminemia among patients with disseminated cancer remains significantly associated with adverse surgical outcomes, and that lower thresholds may be more predictive of high-risk patients in this subgroup. Future studies to validate these thresholds and to extend these findings to other patient populations are warranted.

## Figures and Tables

**Table 1:** Optimal serum albumin cutoff (g/dL) to predict 30-day postoperative adverse events.

Outcomes	Optimal Serum Albumin Cut-Off (g/dL)
Death	3.4
Cardiac Arrest Requiring CPR	3.5
Myocardial Infarction	3.7
Stroke/CVA	3.7
Reoperation	3.5
Composite Primary Adverse Events	3.5
Superficial Incisional SSI	3.7
Deep Incisional SSI	3.6
Organ/Space SSI	3.5
Wound Disruption	3.6
Pneumonia	3.4
Urinary Tract Infection	3.5
Sepsis	3.3
Septic Shock	3.4
Unplanned Intubation	3.5
On Ventilator greater than 48 Hours	3.4
Pulmonary Embolism	3.6
DVT Requiring Therapy	3.2
Acute Renal Failure	3.5
Transfusions	3.3
Unplanned Readmission	3.8
Prolonged length of stay	3.3
Composite Secondary Adverse Events	3.3

CPR—cardiopulmonary resuscitation; CVA—cerebrovascular accident; SSI—surgical site infection; DVT—deep venous thrombosis.

**Table 2:** Baseline Characteristics of Multidisciplinary Cohort Stratified by Preoperative Serum Albumin Concentration.

Characteristic	Serum Albumin > 3.4 g/dL	Serum Albumin ≤ 3.4 g/dL	*p*-Value
	*n* = 694,194	*n* = 148,478	
Age (years), median (IQR)	62.0 (52.0–71.0)	67.0 (56.0–76.0)	<0.0001
Age (years)			<0.0001
0–39	56,369 (8.1)	9,289 (6.3)	
40–49	87,807 (12.6)	12,562 (8.5)	
50–59	154,411 (22.2)	25,906 (17.4)	
60–69	197,460 (28.4)	38,417 (25.9)	
70–79	142,139 (20.5)	35,893 (24.2)	
80+	56,008 (8.1)	26,411 (17.8)	
Race			<0.0001
White	551,786 (79.5)	108,504 (73.1)	
Black	69,489 (10.0)	24,318 (16.4)	
Other	72,919 (10.5)	15,656 (10.5)	
Sex			<0.0001
Female	412,624 (59.4)	75,555 (50.9)	
Male	281,570 (40.6)	72,923 (49.1)	
BMI (kg/m^2^), median (IQR)	30.0 (25.6–36.1)	26.7 (22.8–32.1)	<0.0001
BMI (kg/m^2^)			<0.0001
<18.5	9892 (1.4)	8380 (5.6)	
18.5–24.9	141,294 (20.4)	49,790 (33.5)	
25–29.9	198,005 (28.5)	41,501 (28.0)	
30+	345,003 (49.7)	48,807 (32.9)	
Functional Health Status			<0.0001
Independent	675,984 (97.4)	119,529 (80.5)	
Partially Dependent	13,695 (2.0)	19,585 (13.2)	
Totally Dependent	2349 (0.3)	8396 (5.7)	
Missing	2166 (0.3)	968 (0.7)	
ASA classification			<0.0001
1-No Disturb	15,174 (2.2)	642 (0.4)	
2-Mild Disturb	278,940 (40.2)	20,998 (14.1)	
3-Severe Disturb	351,891 (50.7)	80,360 (54.1)	
4-Life Threat	46,859 (6.8)	43,297 (29.2)	
5-Moribund	1330 (0.2)	3181 (2.1)	
Operation Time (min), median (IQR)	124.0 (85.0–197.0)	136.0 (85.0–218.0)	<0.0001
Preoperative Transfusion *			<0.0001
No	690,325 (99.4)	135,963 (91.6)	
Yes	3868 (0.6)	12,515 (8.4)	
Bleeding disorder			<0.0001
No	666,579 (96.0)	126,745 (85.4)	
Yes	27,615 (4.0)	21,733 (14.6)	
Disseminated cancer			<0.0001
No	666,606 (96.0)	137,235 (92.4)	
Yes	27,588 (4.0)	11,243 (7.6)	
Currently on dialysis *			<0.0001
No	689,092 (99.3)	137,426 (92.6)	
Yes	5101 (0.7)	11,051 (7.4)	
Preoperative acute renal failure *			<0.0001
No	692,761 (99.8)	143,761 (96.8)	
Yes	1432 (0.2)	4717 (3.2)	
Congestive heart failure			<0.0001
No	688,233 (99.1)	140,351 (94.5)	
Yes	5961 (0.9)	8127 (5.5)	
Ascites *			<0.0001
No	692,174 (99.7)	143,836 (96.9)	
Yes	2019 (0.3)	4642 (3.1)	
Preoperative Sepsis*			<0.0001
No	666,181 (96.0)	104,206 (70.2)	
Yes	27,047 (3.9)	44,011 (29.6)	
Ventilator-dependent			<0.0001
No	692,225 (99.7)	141,866 (95.5)	
Yes	1969 (0.3)	6612 (4.5)	
Immunosuppressant use			<0.0001
No	665,891 (95.9)	132,166 (89.0)	
Yes	28,303 (4.1)	16,312 (11.0)	
COPD			<0.0001
No	660,162 (95.1)	131,775 (88.8)	
Yes	34,032 (4.9)	16,703 (11.2)	
Hypertension requiring medication			<0.0001
No	305,244 (44.0)	54,597 (36.8)	
Yes	388,950 (56.0)	93,881 (63.2)	
Active Smoker			<0.0001
No	581,980 (83.8)	113,687 (76.6)	
Yes	112,214 (16.2)	34,791 (23.4)	
Diabetes Mellitus Requiring Therapy			<0.0001
No	565,499 (81.5)	104,211 (70.2)	
Yes	128,695 (18.5)	44,267 (29.8)	
Surgical Specialty			<0.0001
Cardiac Surgery	16,184 (2.3)	3822 (2.6)	
General Surgery	300,387 (43.3)	96,822 (65.2)	
Gynecology	72,119 (10.4)	2457 (1.7)	
Neurosurgery	3249 (0.5)	1094 (0.7)	
Orthopedics	236,067 (34.0)	7415 (5.0)	
Thoracic	13,035 (1.9)	1131 (0.8)	
Urology	19,431 (2.8)	3492 (2.4)	
Vascular	33,722 (4.9)	32,245 (21.7)	

IQR—Interquartile Range; BMI—body mass index; ASA—American Society of Anesthesiologists; COPD—chronic obstructive pulmonary disease. * One patient with missing data.

**Table 3:** Crude Rates of Surgical Outcomes.

Outcomes	*n*	Serum Albumin > 3.4 g/dL	Serum Albumin ≤ 3.4 g/dL	*p*-Value
Death	842,672			<0.0001
No		687,007 (99.0)	134,288 (90.4)	
Yes		7187 (1.0)	14,190 (9.6)	
Cardiac Arrest Requiring CPR	842,672			<0.0001
No		691,126 (99.6)	144,870 (97.6)	
Yes		3068 (0.4)	3608 (2.4)	
Myocardial Infarction	842,672			<0.0001
No		690,943 (99.5)	146,337 (98.6)	
Yes		3251 (0.5)	2141 (1.4)	
Stroke/CVA	842,672			<0.0001
No		692,581 (99.8)	147,235 (99.2)	
Yes		1613 (0.2)	1243 (0.8)	
Reoperation	657,597			<0.0001
No		534,694 (96.6)	94,758 (90.9)	
Yes		18,660 (3.4)	9485 (9.1)	
Composite Primary Adverse Events	842,672			<0.0001
No		665,379 (95.8)	123,593 (83.2)	
Yes		28,815 (4.2)	24,885 (16.8)	
Superficial Incisional SSI	842,672			<0.0001
No		675,126 (97.3)	140,734 (94.8)	
Yes		19,068 (2.7)	7744 (5.2)	
Deep Incisional SSI	842,672			<0.0001
No		689,608 (99.3)	145,658 (98.1)	
Yes		4586 (0.7)	2820 (1.9)	
Organ/Space SSI	842,672			<0.0001
No		677,543 (97.6)	139,270 (93.8)	
Yes		16,651 (2.4)	9208 (6.2)	
Wound Disruption	842,672			<0.0001
No		689,764 (99.4)	145,330 (97.9)	
Yes		4430 (0.6)	3148 (2.1)	
Pneumonia	842,672			<0.0001
No		683,009 (98.4)	137,866 (92.9)	
Yes		11,185 (1.6)	10,612 (7.1)	
Urinary Tract Infection	842,672			<0.0001
No		681,823 (98.2)	142,711 (96.1)	
Yes		12,371 (1.8)	5767 (3.9)	
Sepsis	842,672			<0.0001
No		677,644 (97.6)	135,075 (91.0)	
Yes		16,550 (2.4)	13,403 (9.0)	
Septic Shock	842,672			<0.0001
No		686,127 (98.8)	135,416 (91.2)	
Yes		8067 (1.2)	13,062 (8.8)	
Unplanned Intubation	842,672			<0.0001
No		685,759 (98.8)	140,082 (94.3)	
Yes		8435 (1.2)	8396 (5.7)	
On ventilator greater than 48 Hours	842,672			<0.0001
No		683,240 (98.4)	132,297 (89.1)	
Yes		10,954 (1.6)	16,181 (10.9)	
Pulmonary Embolism	842,672			<0.0001
No		690,496 (99.5)	146,892 (98.9)	
Yes		3698 (0.5)	1586 (1.1)	
DVT Requiring Therapy	842,672			<0.0001
No		687,672 (99.1)	144,065 (97.0)	
Yes		6522 (0.9)	4413 (3.0)	
Acute Renal Failure	842,672			<0.0001
No		691,259 (99.6)	144,828 (97.5)	
Yes		2935 (0.4)	3650 (2.5)	
Transfusions	842,672			<0.0001
No		636,352 (91.7)	109,523 (73.8)	
Yes		57,842 (8.3)	38,955 (26.2)	
Unplanned Readmission	656,377			<0.0001
No		514,922 (93.2)	90,290 (86.7)	
Yes		37,287 (6.8)	13,878 (13.3)	
Prolonged length of stay	840,857			<0.0001
No		585,042 (84.4)	90,970 (61.7)	
Yes		108,294 (15.6)	56,551 (38.3)	
Composite Secondary Adverse Events	842,672			<0.0001
No		488,394 (70.4)	50,783 (34.2)	
Yes		205,800 (29.6)	97,695 (65.8)	

CPR—cardiopulmonary resuscitation; CVA—cerebrovascular accident; SSI—surgical site infection; DVT—deep venous thrombosis.

**Table 4:** Subgroup Analysis—The effect of hypoalbuminemia on adverse surgical events across surgical specialties (results from a multivariable logistic regression model, presented as odds ratios with 95% confidence intervals).

**OR (95% CI)**	**Cardiac Surgery**	**General Surgery**	**Gynecology**	**Neurosurgery**	**Orthopedics**	**Thoracic**	**Urology**	**Vascular**
Death	1.80 (1.49–2.17)	2.18 (2.08–2.27)	5.11 (2.44–10.74)	1.29 (1.06–1.56)	3.50 (2.71–4.52)	2.42 (1.74–3.36)	2.07 (1.60–2.68)	1.52 (1.40–1.65)
Cardiac Arrest Requiring CPR	1.23 (1.00–1.51)	1.53 (1.42–1.65)	2.81 (0.96–8.24)	1.28 (0.79–2.08)	1.56 (0.99–2.47)	2.63 (1.70–4.04)	1.41 (0.97–2.05)	1.32 (1.17–1.50)
Myocardial Infarction	0.91 (0.57–1.45)	1.11 (1.02–1.21)	0.34 (0.04–2.64)	1.00 (0.49–2.01)	1.57 (1.13–2.20)	0.93 (0.42–2.05)	1.06 (0.73–1.55)	0.97 (0.85–1.09)
Stroke/CVA	1.16 (0.91–1.49)	1.55 (1.37–1.76)	3.77 (0.81–17.64)	1.30 (0.87–1.94)	1.76 (1.08–2.86)	0.96 (0.44–2.13)	1.89 (1.10–3.26)	1.00 (0.82–1.22)
Reoperation	1.02 (0.86–1.21)	1.35 (1.30–1.41)	1.10 (0.78–1.54)	0.90 (0.72–1.12)	1.81 (1.57–2.07)	1.73 (1.33–2.23)	1.02 (0.82–1.27)	1.24 (1.16–1.33)
Composite Primary Adverse Events	1.29 (1.15–1.45)	1.58 (1.54–1.63)	1.32 (0.99–1.76)	1.21 (1.03–1.41)	2.05 (1.83–2.29)	1.86 (1.52–2.27)	1.39 (1.19–1.63)	1.32 (1.25–1.39)
Superficial Incisional SSI	1.08 (0.84–1.39)	1.24 (1.20–1.29)	1.54 (1.07–2.21)	1.51 (0.53–4.36)	1.48 (1.15–1.91)	1.14 (0.58–2.24)	1.32 (1.05–1.65)	1.00 (0.92–1.09)
Deep Incisional SSI	1.40 (0.84–2.33)	1.59 (1.49–1.70)	1.22 (0.60–2.50)	0.22 (0.03–1.81)	2.21 (1.56–3.12)	0.63 (0.08–5.08)	1.06 (0.63–1.77)	1.13 (1.00–1.28)
Organ/Space SSI	1.24 (0.65–2.36)	1.50 (1.45–1.55)	1.47 (1.05–2.05)	1.20 (0.51–2.82)	2.27 (1.64–3.13)	1.04 (0.55–1.95)	1.65 (1.35–2.02)	1.42 (1.18–1.72)
Wound Disruption	1.19 (0.75–1.91)	1.64 (1.54–1.75)	1.23 (0.58–2.61)	0.30 (0.03–3.06)	1.34 (0.89–2.02)	*	1.20 (0.82–1.76)	1.15 (1.00–1.33)
Pneumonia	1.25 (1.06–1.46)	1.52 (1.46–1.58)	3.50 (2.15–5.69)	1.03 (0.83–1.28)	2.79 (2.29–3.40)	1.73 (1.41–2.14)	1.42 (1.13–1.78)	1.22 (1.11–1.33)
Urinary Tract Infection	1.34 (1.03–1.74)	1.34 (1.28–1.40)	1.11 (0.86–1.42)	1.21 (0.90–1.63)	1.47 (1.22–1.76)	1.82 (1.18–2.80)	0.95 (0.78–1.17)	1.26 (1.13–1.42)
Sepsis	1.57 (1.23–2.02)	1.60 (1.55–1.65)	1.77 (1.16–2.70)	0.96 (0.69–1.32)	2.93 (2.29–3.75)	2.68 (1.90–3.76)	1.19 (0.98–1.44)	1.43 (1.31–1.57)
Septic Shock	1.73 (1.33–2.25)	1.75 (1.68–1.81)	5.45 (2.86–10.38)	2.04 (1.34–3.11)	2.83 (1.82–4.41)	2.26 (1.52–3.35)	1.92 (1.48–2.51)	1.65 (1.48–1.85)
Unplanned Intubation	1.45 (1.22–1.71)	1.68 (1.61–1.76)	3.17 (1.80–5.60)	0.97 (0.74–1.26)	2.36 (1.76–3.17)	1.99 (1.55–2.54)	1.30 (1.02–1.66)	1.10 (1.00–1.21)
On Ventilator greater than 48 Hours	1.61 (1.41–1.84)	1.84 (1.77–1.91)	4.31 (2.08–8.97)	1.09 (0.90–1.31)	3.06 (2.07–4.51)	2.27 (1.73–2.97)	1.81 (1.39–2.36)	1.12 (1.03–1.23)
Pulmonary Embolism	1.11 (0.66–1.86)	1.54 (1.42–1.67)	1.35 (0.70–2.62)	1.18 (0.63–2.22)	1.42 (1.06–1.91)	1.33 (0.70–2.52)	1.60 (1.16–2.22)	1.27 (0.96–1.68)
DVT Requiring Therapy	1.37 (1.05–1.79)	1.90 (1.80–2.01)	1.03 (0.46–2.31)	1.53 (1.12–2.10)	1.23 (0.97–1.56)	1.32 (0.80–2.17)	1.65 (1.26–2.17)	1.33 (1.13–1.56)
Acute Renal Failure	2.11 (1.71–2.60)	1.82 (1.69–1.97)	3.72 (1.30–10.65)	1.44 (0.68–3.04)	2.28 (1.35–3.86)	2.46 (1.35–4.51)	1.29 (0.95–1.76)	1.38 (1.21–1.57)
Transfusions	1.32 (1.22–1.43)	2.48 (2.42–2.54)	2.80 (2.31–3.39)	1.74 (1.42–2.14)	1.78 (1.66–1.92)	2.66 (2.21–3.21)	2.59 (2.37–2.84)	1.53 (1.46–1.60)
Unplanned Readmission	1.12 (0.96–1.30)	1.17 (1.14–1.21)	1.61 (1.34–1.95)	1.14 (0.87–1.50)	1.70 (1.54–1.87)	1.19 (0.94–1.52)	1.07 (0.94–1.21)	1.18 (1.10–1.25)
Prolonged length of stay	2.46 (2.26–2.68)	2.34 (2.30–2.38)	2.18 (1.97–2.40)	1.64 (1.39–1.94)	2.51 (2.37–2.64)	2.48 (2.15–2.85)	2.38 (2.18–2.60)	2.51 (2.40–2.63)
Composite Secondary Adverse Events	1.84 (1.67–2.03)	2.31 (2.27–2.36)	2.05 (1.87–2.25)	1.52 (1.29–1.80)	2.32 (2.20–2.44)	2.65 (2.31–3.05)	2.59 (2.38–2.83)	2.01 (1.93–2.09)

OR—odds ratio, CI—confidence interval; CPR—cardiopulmonary resuscitation; CVA—cerebrovascular accident; SSI—surgical site infection; DVT—deep venous thrombosis. * Patients with thoracic specialty had skewed distribution in Wound Disruption outcome. Logistic regression model could not be fitted.

## Data Availability

The data are not publicly available due to legal restrictions.

## Supplementary Materials

The following supporting information can be downloaded at: https://www.mdpi.com/article/10.3390/jcm11216543/s1, Figure S1: Relative Risk of Selected Adverse Events Stratified by Serum Albumin, Derived from Unadjusted Crude Rates; Figure S2: Odds of composite and primary adverse events according to hypoalbuminemia; Figure S3: Odds of selected secondary adverse events stratified by serum albumin, adjusted for relevant clinical covariates; Figure S4: Odds of adverse events in individual specialties, stratified by serum albumin, adjusted for relevant clinical covariates; Table S1: CPT codes used to generate multi-surgical cohort, stratified by surgical specialty; Table S2: Optimal serum albumin cutoff (g/dL) to predict 30-day postoperative adverse events, stratified by disseminated cancer status; Table S3: Subgroup Analysis—Impact of Disseminated Cancer on Crude Rates of Adverse Surgical Outcomes Stratified by Serum Albumin; Table S4: Multivariable Logistic Regression Model. Results presented as odds ratio (95% CI); Table S5: Subgroup Analysis—Impact of Disseminated Cancer in Multivariable Logistic Regression Model; Table S6: Spline Analysis.

## References

[1] Bing J., Naeser J., Rasch G., Røjel K. (2009). Serum Proteins in Normal People. Acta Med. Scand..

[2] Arques S. (2018). Human serum albumin in cardiovascular diseases. Eur. J. Intern. Med..

[3] D’Amico G., Garcia-Tsao G., Pagliaro L. (2006). Natural history and prognostic indicators of survival in cirrhosis: A systematic review of 118 studies. J. Hepatol..

[4] Morotti A., Marini S., Lena U.K., Crawford K., Schwab K., Kourkoulis C., Ayres A.M., Edip G.M., Viswanathan A., Greenberg S.M. (2017). Significance of admission hypoalbuminemia in acute intracerebral hemorrhage. J. Neurol..

[5] Zhou H., Wang A., Meng X., Lin J., Jiang Y., Jing J., Zuo Y., Wang Y., Zhao X., Li H. (2021). Low serum albumin levels predict poor outcome in patients with acute ischaemic stroke or transient ischaemic attack. Stroke Vasc. Neurol..

[6] Alves F.C., Sun J., Qureshi A.R., Dai L., Snaedal S., Barany P., Heimburger O., Lindholm B., Stenvinkel P. (2018). The higher mortality associated with low serum albumin is dependent on systemic inflammation in end-stage kidney disease. PLoS ONE.

[7] Zhang J., Zhang R., Wang Y., Li H., Han Q., Wu Y., Wang T., Liu F. (2019). The Level of Serum Albumin Is Associated with Renal Prognosis in Patients with Diabetic Nephropathy. J. Diabetes Res..

[8] Seo M.H., Choa M., You J.S., Lee H.S., Hong J.H., Park Y.S., Chung S.P., Park I. (2016). Hypoalbuminemia, Low Base Excess Values, and Tachypnea Predict 28-Day Mortality in Severe Sepsis and Septic Shock Patients in the Emergency Department. Yonsei Med. J..

[9] Lomholt F.K., Laulund A.S., Bjarnason N.H., Jørgensen H.L., Godtfredsen N.S. (2014). Meta-analysis of routine blood tests as predictors of mortality in COPD. Eur. Clin. Respir. J.

[10] Huang J., Cheng A., Kumar R., Fang Y., Chen G., Zhu Y., Lin S. (2020). Hypoalbuminemia predicts the outcome of COVID-19 independent of age and co-morbidity. J. Med. Virol..

[11] Thongprayoon C., Cheungpasitporn W., Chewcharat A., Mao M.A., Thirunavukkarasu S., Kashani K.B. (2019). Impacts of admission serum albumin levels on short-term and long-term mortality in hospitalized patients. QJM.

[12] Leite H.P., da Silva A.V.R., de Oliveira Iglesias S.B., Nogueira P.C.K. (2016). Serum Albumin Is an Independent Predictor of Clinical Outcomes in Critically Ill Children. Pediatr. Crit. Care Med..

[13] Herrmann F.R., Safran C., Levkoff S.E., Minaker K.L. (1992). Serum Albumin Level on Admission as a Predictor of Death, Length of Stay, and Readmission. Arch. Intern. Med..

[14] Adogwa O., Martin J.R., Huang K., Verla T., Fatemi P., Thompson P., Cheng J., Kuchibhatla M., Lad S.P., Bagley C.A. (2014). Preoperative Serum Albumin Level as a Predictor of Postoperative Complication After Spine Fusion. Spine.

[15] Larsen P.B., Liest S., Hannani D., Jørgensen H.L., Sørensen L.T., Jørgensen L.N. (2019). Preoperative Hypoalbuminemia Predicts Early Mortality Following Open Abdominal Surgery in Patients Above 60 Years of Age. Scand. J. Surg..

[16] Rapp-Kesek D., Ståhle E., Karlsson T.T. (2004). Body mass index and albumin in the preoperative evaluation of cardiac surgery patients. Clin. Nutr..

[17] Neel D.R., McClave S., Martindale R. (2011). Hypoalbuminaemia in the perioperative period: Clinical significance and management options. Best Pract. Res. Clin. Anaesthesiol..

[18] Gibbs J., Cull W., Henderson W., Daley J., Hur K., Khuri S.F. (1999). Preoperative Serum Albumin Level as a Predictor of Operative Mortality and Morbidity. Arch. Surg..

[19] Don B.R., Kaysen G. (2004). Poor Nutritional Status and Inflammation: Serum Albumin: Relationship to Inflammation and Nutrition. Semin. Dial..

[20] Huang Y., Alzahrani N.A., Chua T.C., Huo Y.R., Liauw W., Morris D.L. (2016). Impacts of Preoperative Serum Albumin Level on Outcomes of Cytoreductive Surgery and Perioperative Intraperitoneal Chemotherapy. Ann. Surg. Oncol..

[21] Ataseven B., du Bois A., Reinthaller A., Traut A., Heitz F., Aust S., Prader S., Polterauer S., Harter P., Grimm C. (2015). Pre-operative serum albumin is associated with post-operative complication rate and overall survival in patients with epithelial ovarian cancer undergoing cytoreductive surgery. Gynecol. Oncol..

[22] Margarson M.P., Soni N. (1998). Serum albumin: Touchstone or totem?. Anaesthesia.

[23] Mullen J.L., Gertner M.H., Buzby G.P., Goodhart G.L., Rosato E.F. (1979). Implications of Malnutrition in the Surgical Patient. Arch. Surg..

[24] Curran S., Apruzzese P., Kendall M.C., De Oliveira G. (2022). The impact of hypoalbuminemia on postoperative outcomes after outpatient surgery: A national analysis of the NSQIP database. Can. J. Anaesth..

[25] Goldwasser P., Feldman J. (1997). Association of serum albumin and mortality risk. J. Clin. Epidemiol..

[26] Caraceni P., Tufoni M., Bonavita M.E. (2013). Clinical use of albumin. Blood Transfus..

[27] Liumbruno G.M., Bennardello F., Lattanzio A., Piccoli P., Rossetti G. (2009). Recommendations for the use of albumin and immunoglobulins. Blood Transfus..

[28] Roberts I., Blackhall K., Alderson P., Bunn F., Schierhout G. (2011). Human albumin solution for resuscitation and volume expansion in critically ill patients. Cochrane Database Syst. Rev..

[29] Hubner M., Mantziari S., Demartines N., Pralong F., Coti-Bertrand P., Schafer M. (2016). Postoperative Albumin Drop Is a Marker for Surgical Stress and a Predictor for Clinical Outcome: A Pilot Study. Gastroenterol. Res. Pract..

[30] Loftus T.J., Brown M.P., Slish J.H., Rosenthal M.D. (2019). Serum Levels of Prealbumin and Albumin for Preoperative Risk Stratification. Nutr. Clin. Pract..

