# Supplementary material for: The Association between Serum Albumin and Post-Operative Outcomes among Patients Undergoing Common Surgical Procedures: An Analysis of a Multi-Specialty Surgical Cohort from the National Surgical Quality Improvement Program (NSQIP)
Source: J Clin Med. 2022 Nov 4;11(21):6543. doi: 10.3390/jcm11216543 (PMC9655694; doi:10.3390/jcm11216543)
Supplement: Supplementary file 1 [file jcm-11-06543-s001.zip › jcm-1938355-supplementary.pdf]

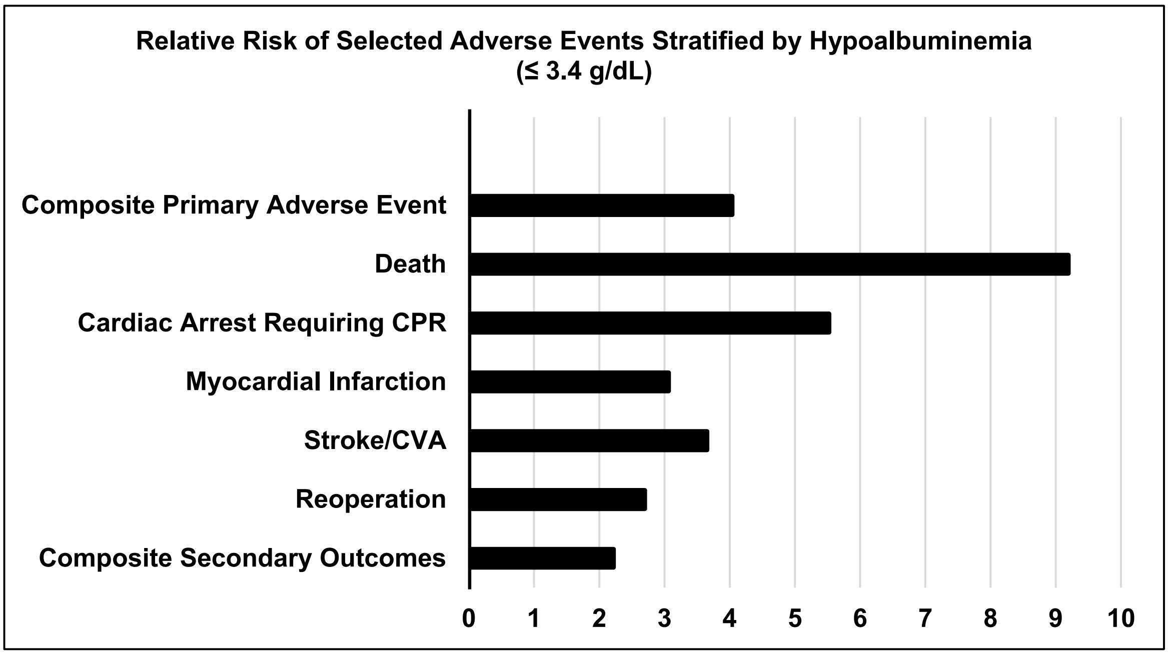

Figure S1. Relative Risk of Selected Adverse Events Stratified by Serum Albumin, Derived from Unadjusted Crude Rates–
Hypoalbuminemia increases unadjusted relative risk of selected adverse events. CPR – cardiopulmonary resuscitation; CVA – cerebrovascular accident.

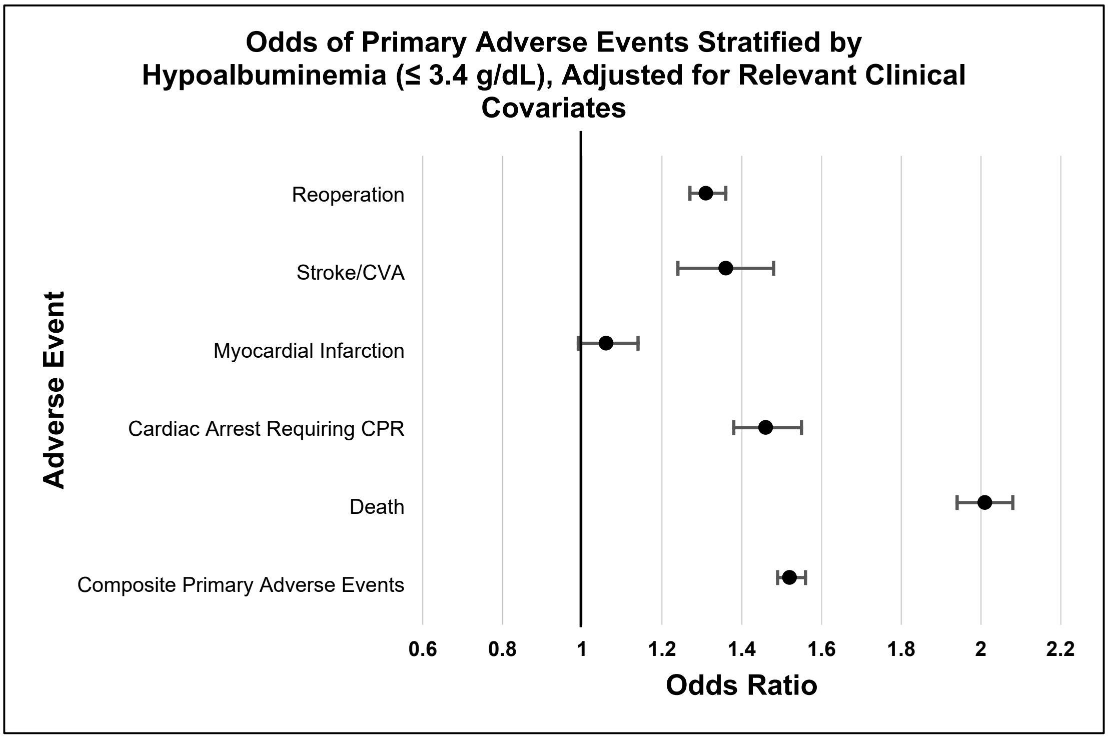

Figure S2. Odds of composite and primary adverse events according to hypoalbuminemia – Hypoalbuminemia increases odds of all primary outcomes except myocardial infarction after multivariate adjustment. The composite odds of primary adverse events are also increased in patients with hypoalbuminemia. CVA – cerebrovascular accident; CPR – cardiopulmonary resuscitation.

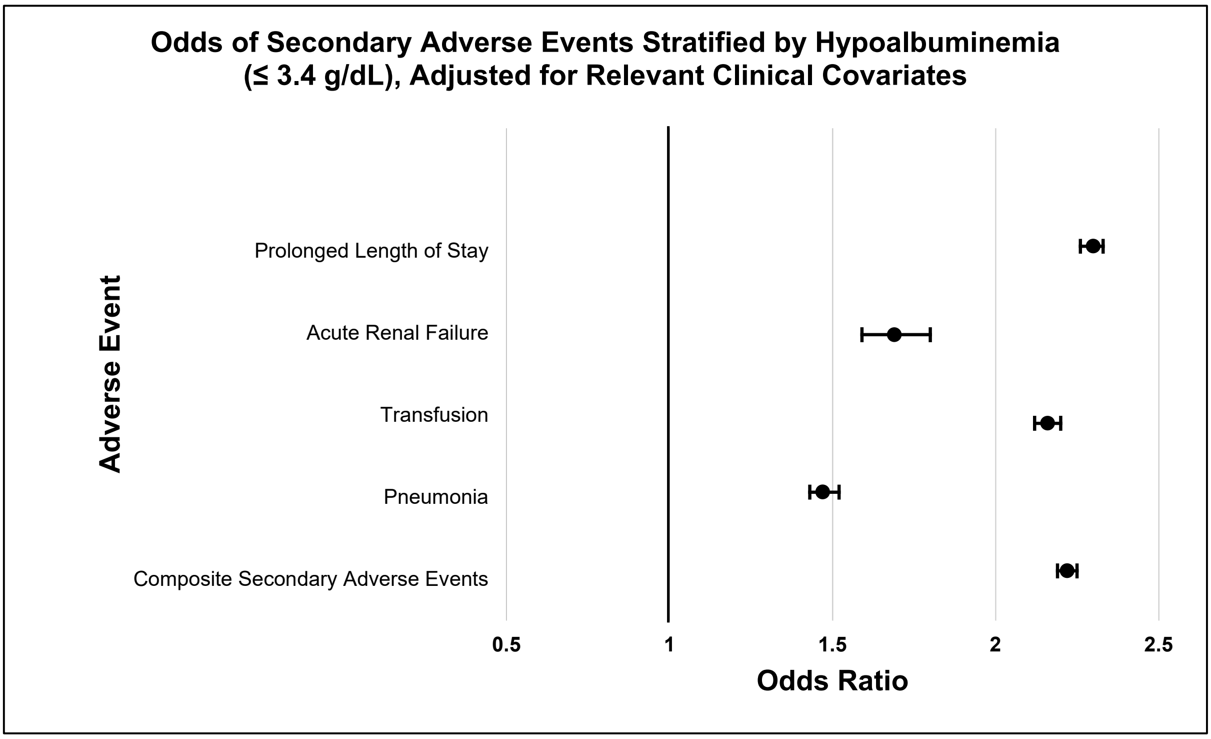

Figure S3. Odds of selected secondary adverse events stratified by serum albumin, adjusted for relevant clinical covariates–
Hypoalbuminemia increases odds of all secondary adverse outcomes after multivariate adjustment. The composite odds of secondary adverse events are also increased in patients with hypoalbuminemia.

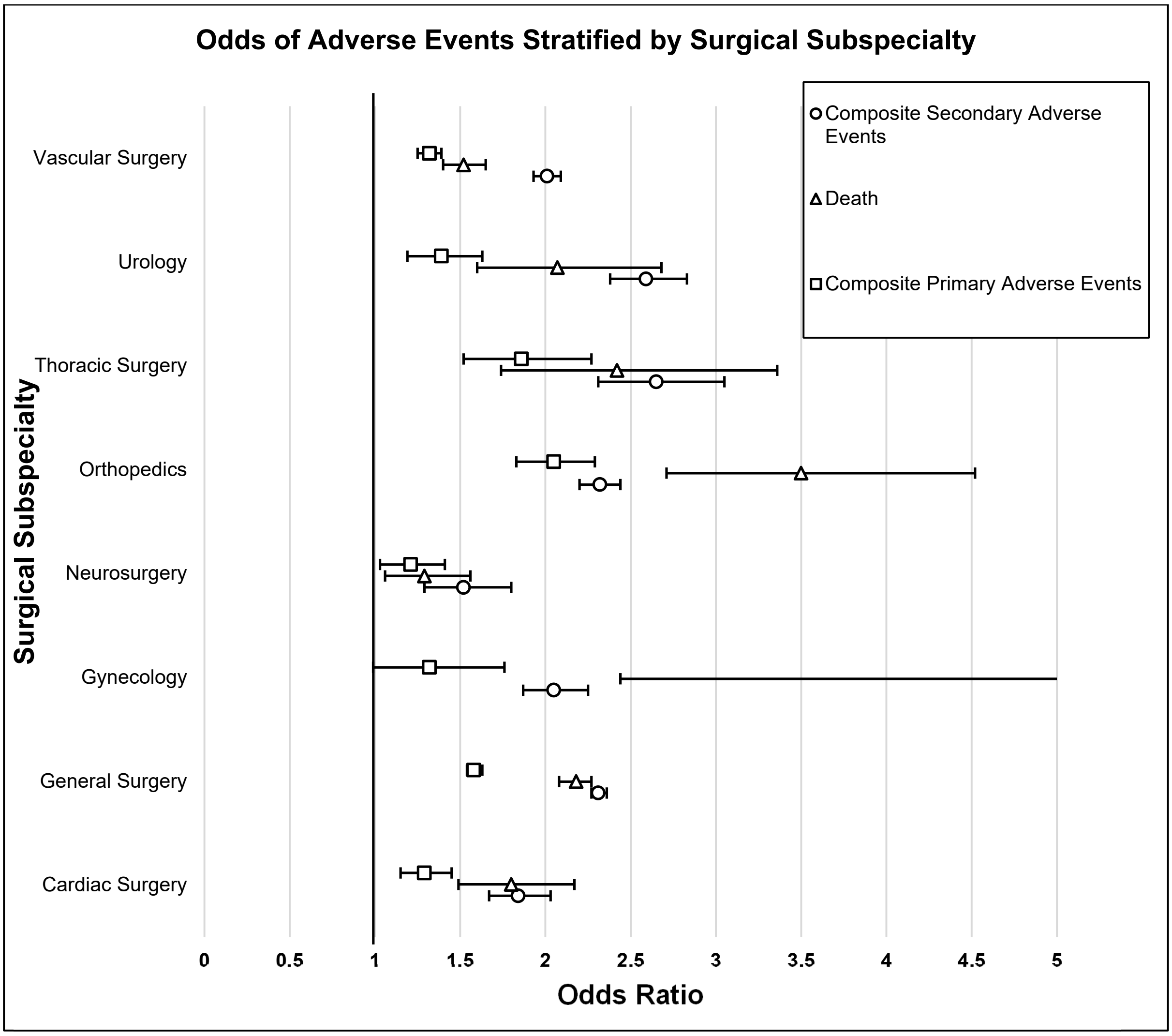

Figure S4. Odds of adverse events in individual specialties, stratified by serum albumin, adjusted for relevant clinical covariates
Hypoalbuminemia increases odds of composite odds of primary and secondary adverse events in the majority of surgical subspecialties.

Table S1. CPT codes used to generate multi-surgical cohort, stratified by surgical specialty

Description	CPT codes
**Vascular Surgery**	
**Open abdominal aortic aneurysm repair**	34830, 34831, 34832, 35081, 35082, 35091, 35092, 35102, 35103
**Endovascular abdominal aortic aneurysm repair**	34701, 34702, 34703, 34704, 34705, 34706
**Below knee amputation**	27880, 27882, 27881
**Above knee amputation**	27592, 27590, 27591
**Peripheral vascular surgery (Artery and vein bypass procedures: aorto-bifemoral, aorto-femoral, ileo-femoral, femoral-popliteal, femoral-tibial, aorto-femoral-popliteal, femoral-femoral)**	35551, 35646, 35647, 35666, 35665, 35540, 35539, 35566, 35565, 35585, 35656, 35556, 35583, 35548, 35661, 35558
**General Surgery**	
**Large bowel/rectal surgery**	44150, 44151, 44155, 44156, 44157, 44158, 44211(r), 44212, 44210, 44120, 44204, 44206, 44207, 44208, 44140, 44141, 44143, 44144, 44145, 44146, 45110, 45112, 45119, 45120, 45121, 45395, 45397
**Hepatic/Liver resection**	47379, 47120(r), 47122, 47125, 47130
**Whipple's pancreaticoduodenectomy**	48999, 48150, 48152, 48153, 48154
**Gastric bypass**	43846, 43847, 43644r, 43645(r)
**Thoracic Surgery**	
**Open pneumonectomy or lobectomy**	32440, 32442, 32445, 32488, 32482, 32480, 32484, 32486, 32491
**VATS lobectomy**	32491T, 32663
**Urology**	
**Radical Cystectomy**	51570, 51590(r), 51575, 51596(r), 51580, 51570, 51585, 51595
**Nephrectomy**	50225, 50230, 50220, 50545(r), 50546
**Nephroureterectomy**	50234(r), 50236(r)
**Orthopedic Surgery**	
**Total hip and knee joint replacement**	27130, 27447, 27445
**Spinal surgery (Multisegment surgery, cervical arthroplasty/replacement)**	22845 22846 22847<br>22856 22858<br>22840 22841 22842 22843 22844<br>63050 63051<br>22845 22846 22847<br>22845 22846 22847<br>22840 22841 22842 22843 22844<br>22840 22841 22842 22843 22844<br>22840 22841 22842 22843 22844
**Hip fixation**	27253, 27254, 27259
**Neurosurgery**	

**Open craniotomy, craniectomy, posterior fossa surgery**	61304, 61305, 61522, 61312, 61313, 61314, 61315, 61322, 61323
**Cardiac Surgery**	
**Coronary artery bypass grafting**	33510, 33511, 33512, 33514, 33515, 33516, 33517, 33518, 33519, 33520, 33521, 33522, 33523, 33533, 33534, 33535, 33536
**Single valve repair or replacement**	33602, 33391, 33390, 33600, 33425, 33426, 33427, 33420, 33422, 33470, 33471, 33474, 33468, 33463, 33464, 33430, 33465, 33475, 33405, 33406, 33410
**Complex: multiple valve surgeries, aortic procedures, ventricular aneurysmectomy, assist device**	33866, 33863, 33413, 33440, 33412, 33411, 33548, 33542, 33975, 33979, 33622
**Gynecology**	
**Hysterectomy**	58152, 58541-58544, 58260-58294, 58570, 58571, 58572, 58573, 58550, 58552, 58553, 58554, 58542, 58541, 58543, 58544

Table S2. Optimal serum albumin cutoff (g/dL) to predict 30-day postoperative adverse events, stratified by disseminated cancer status

Outcomes	Disseminated Cancer Absent	Disseminated Cancer Present
**Composite Primary Adverse Event**	3.5	3.0
**Death**	3.5	3.1
**Cardiac Arrest Requiring CPR**	3.5	3.1
**Myocardial Infarction**	3.9	1.5
**Stroke/CVA**	3.8	3.2
**Reoperation**	3.5	3.8

CPR – cardiopulmonary resuscitation; CVA – cerebrovascular accident.

Table S3. Subgroup Analysis – Impact of Disseminated Cancer on Crude Rates of Adverse Surgical Outcomes Stratified by Serum Albumin

	N	Serum Albumin >	Serum Albumin ≤	p-value
**Death**	38,831			
**No**		36,354 (93.6)	26,958 (97.7)	9,396
**Yes**		2,477 (6.4)	630 (2.3)	1,847
**Cardiac Arrest Requiring**	38,831			
**No**		38,437 (99.0)	27,408 (99.3)	11,029
**Yes**		394 (1.0)	180 (0.7)	214 (1.9)
**Myocardial Infarction**	38,831			
**No**		38,520 (99.2)	27,401 (99.3)	11,119
**Yes**		311 (0.8)	187 (0.7)	124 (1.1)
**Stroke/CVA**	38,831			
**No**		38,654 (99.5)	27,506 (99.7)	11,148
**Yes**		177 (0.5)	82 (0.3)	95 (0.8)
**Reoperation**	30,343			
**No**		28,602 (94.3)	20,785 (95.1)	7,817
**Yes**		1,741 (5.7)	1,060 (4.9)	681 (8.0)
**Composite Primary**	38,831			
**No**		34,503 (88.9)	25,774 (93.4)	8,729
**Yes**		4,328 (11.1)	1,814 (6.6)	2,514
**Superficial Incisional SSI**	38,831			
**No**		36,951 (95.2)	26,392 (95.7)	10,559
**Yes**		1,880 (4.8)	1,196 (4.3)	684 (6.1)
**Deep Incisional SSI**	38,831			
**No**		38,286 (98.6)	27,272 (98.9)	11,014
**Yes**		545 (1.4)	316 (1.1)	229 (2.0)
**Organ/Space SSI**	38,831			
**No**		36,054 (92.8)	25,836 (93.6)	10,218
**Yes**		2,777 (7.2)	1,752 (6.4)	1,025
**Wound Disruption**	38,831			
**No**		38,247 (98.5)	27,265 (98.8)	10,982
**Yes**		584 (1.5)	323 (1.2)	261 (2.3)
**Pneumonia**	38,831			
**No**		37,116 (95.6)	26,753 (97.0)	10,363
**Yes**		1,715 (4.4)	835 (3.0)	880 (7.8)
**Urinary Tract Infection**	38,831			
**No**		37,568 (96.7)	26,802 (97.2)	10,766
**Yes**		1,263 (3.3)	786 (2.8)	477 (4.2)
**Sepsis**	38,831			
**No**		36,201 (93.2)	26,175 (94.9)	10,026
**Yes**		2,630 (6.8)	1,413 (5.1)	1,217
**Septic Shock**	38,831			
**No**		37,056 (95.4)	26,949 (97.7)	10,107
**Yes**		1,775 (4.6)	639 (2.3)	1,136

**Unplanned Intubation**	38,831			
**No**		37,548 (96.7)	26,980 (97.8)	10,568
**Yes**		1,283 (3.3)	608 (2.2)	675 (6.0)
**On Ventilator greater than**	38,831			
**No**		37,079 (95.5)	26,944 (97.7)	10,135
**Yes**		1,752 (4.5)	644 (2.3)	1,108
**Pulmonary Embolism**	38,831			
**No**		38,267 (98.5)	27,263 (98.8)	11,004
**Yes**		564 (1.5)	325 (1.2)	239 (2.1)
**DVT Requiring Therapy**	38,831			
**No**		37,872 (97.5)	27,096 (98.2)	10,776
**Yes**		959 (2.5)	492 (1.8)	467 (4.2)
**Acute Renal Failure**	38,831			
**No**		38,403 (98.9)	27,403 (99.3)	11,000
**Yes**		428 (1.1)	185 (0.7)	243 (2.2)
**Transfusions**	38,831			
**No**		30,631 (78.9)	23,071 (83.6)	7,560
**Yes**		8,200 (21.1)	4,517 (16.4)	3,683
**Unplanned Readmission**	30,301			
**No**		26,272 (86.7)	19,170 (87.9)	7,102
**Yes**		4,029 (13.3)	2,641 (12.1)	1,388
**Prolonged length of stay**	38,716			
**No**		28,738 (74.2)	22,160 (80.5)	6,578
**Yes**		9,978 (25.8)	5,380 (19.5)	4,598
**Composite Secondary Adverse Events**	38,831			
**No**		19,028 (49.0)	15,905 (57.7)	3,123
**Yes**		19,803 (51.0)	11,683 (42.3)	8,120

CPR – cardiopulmonary resuscitation; CVA – cerebrovascular accident; SSI – surgical site infection; DVT – deep venous thrombosis.

Table S4. Multivariate Logistic Regression Model. Results presented as odds ratio (95% CI)

Outcomes	OR (95%CI)	p-value
**Death**	2.01 (1.94 - 2.08)	<.0001
**Cardiac Arrest Requiring CPR**	1.46 (1.38 - 1.55)	<.0001
**Myocardial Infarction**	1.06 (1.00 - 1.14)	0.0650
**Stroke/CVA**	1.36 (1.24 - 1.48)	<.0001
**Reoperation**	1.31 (1.27 - 1.36)	<.0001
**Composite Primary Adverse Events**	1.52 (1.49 - 1.56)	<.0001
**Superficial Incisional SSI**	1.23 (1.19 - 1.27)	<.0001
**Deep Incisional SSI**	1.52 (1.44 - 1.61)	<.0001
**Organ/Space SSI**	1.51 (1.47 - 1.56)	<.0001
**Wound Disruption**	1.56 (1.48 - 1.65)	<.0001
**Pneumonia**	1.47 (1.43 - 1.52)	<.0001
**Urinary Tract Infection**	1.33 (1.28 - 1.38)	<.0001
**Sepsis**	1.60 (1.56 - 1.65)	<.0001
**Septic Shock**	1.76 (1.70 - 1.82)	<.0001
**Unplanned Intubation**	1.56 (1.50 - 1.62)	<.0001
**On Ventilator greater than 48 Hours**	1.67 (1.62 - 1.72)	<.0001
**Pulmonary Embolism**	1.50 (1.39 - 1.61)	<.0001
**DVT Requiring Therapy**	1.78 (1.70 - 1.87)	<.0001
**Acute Renal Failure**	1.69 (1.59 - 1.80)	<.0001
**Transfusions**	2.16 (2.12 - 2.20)	<.0001
**Unplanned Readmission**	1.20 (1.17 - 1.23)	<.0001
**Prolonged length of stay**	2.30 (2.26 - 2.33)	<.0001
**Composite Secondary Adverse Events**	2.22 (2.19 - 2.25)	<.0001

OR – odds ratio, CI – confidence interval; CPR – cardiopulmonary resuscitation; CVA – cerebrovascular accident; SSI – surgical site infection; DVT – deep venous thrombosis.

Table S5. Subgroup Analysis - Impact of Disseminated Cancer in Multivariate Logistic Regression Model

OR (95%CI)	Disseminated Cancer Absent	Disseminated Cancer Present
**Death**	1.88 (1.81 - 1.96)	3.14 (2.82 - 3.50)
**Cardiac Arrest Requiring CPR**	1.45 (1.36 - 1.54)	1.75 (1.38 - 2.22)
**Myocardial Infarction**	1.07 (1.00 - 1.14)	1.01 (0.77 - 1.32)
**Stroke/CVA**	1.33 (1.21 - 1.46)	1.75 (1.24 - 2.46)
**Reoperation**	1.31 (1.27 - 1.35)	1.35 (1.20 - 1.52)
**Composite Primary Adverse Events**	1.47 (1.44 - 1.51)	2.03 (1.88 - 2.20)
**Superficial Incisional SSI**	1.21 (1.17 - 1.25)	1.49 (1.33 - 1.67)
**Deep Incisional SSI**	1.51 (1.43 - 1.60)	1.62 (1.33 - 1.98)
**Organ/Space SSI**	1.53 (1.48 - 1.58)	1.40 (1.27 - 1.54)
**Wound Disruption**	1.56 (1.48 - 1.66)	1.55 (1.27 - 1.88)
**Pneumonia**	1.47 (1.42 - 1.52)	1.54 (1.37 - 1.73)
**Urinary Tract Infection**	1.34 (1.28 - 1.39)	1.29 (1.13 - 1.48)
**Sepsis**	1.60 (1.56 - 1.65)	1.61 (1.46 - 1.77)
**Septic Shock**	1.76 (1.70 - 1.82)	1.76 (1.55 - 1.98)
**Unplanned Intubation**	1.55 (1.49 - 1.61)	1.78 (1.56 - 2.04)
**On Ventilator greater than 48 Hours**	1.66 (1.60 - 1.71)	1.82 (1.61 - 2.06)
**Pulmonary Embolism**	1.46 (1.36 - 1.58)	1.71 (1.41 - 2.08)
**DVT Requiring Therapy**	1.76 (1.68 - 1.85)	1.87 (1.61 - 2.18)
**Acute Renal Failure**	1.68 (1.58 - 1.79)	1.91 (1.51 - 2.41)
**Transfusions**	2.17 (2.13 - 2.21)	2.08 (1.95 - 2.21)
**Unplanned Readmission**	1.19 (1.16 - 1.22)	1.32 (1.22 - 1.43)
**Prolonged length of stay**	2.28 (2.24 - 2.31)	2.56 (2.42 - 2.71)
**Composite Secondary Adverse Events**	2.19 (2.16 - 2.22)	2.63 (2.48 - 2.78)

OR – odds ratio, CI – confidence interval; CPR – cardiopulmonary resuscitation; CVA – cerebrovascular accident; SSI – surgical site infection; DVT – deep venous thrombosis.

Table S6. Spline Analysis

Serum Albumin Concentration Range (g/dL)	0 to 1.4	1.4 to 2.4	2.4 to 3.4	3.4 to 4.4	4.4 to 5.4	5.4 to 6.4	> 6.4
**N**	**2,916**	**30,526**	**115,036**	**525,095**	**168,077**	**481**	**541**
**Outcomes**	**OR (95%CI)**	**OR (95%CI)**	**OR (95%CI)**	**OR (95%CI)**	**OR (95%CI)**	**OR (95%CI)**	**OR (95%CI)**
Death	0.62 (0.35 - 1.10)	0.63 (0.58 - 0.69)	0.66 (0.62 - 0.70)	0.45 (0.42 - 0.48)	1.25 (0.90 - 1.72)	2.09 (0.89 - 4.91)	1.17 (0.64 - 2.16)
Cardiac Arrest Requiring CPR	1.42 (0.53 - 3.87)	0.71 (0.61 - 0.83)	0.92 (0.83 - 1.02)	0.55 (0.50 - 0.62)	1.04 (0.66 - 1.66)	0.73 (0.14 - 3.74)	2.28 (1.01 - 5.17)
Myocardial Infarction	1.29 (0.24 - 6.92)	0.91 (0.73 - 1.13)	1.20 (1.07 - 1.35)	0.75 (0.67 - 0.83)	0.71 (0.44 - 1.12)	0.65 (0.07 - 6.10)	1.34 (0.30 - 6.00)
Stroke/CVA	0.41 (0.07 - 2.51)	1.10 (0.83 - 1.46)	0.90 (0.77 - 1.06)	0.67 (0.58 - 0.78)	0.67 (0.35 - 1.30)	3.39 (0.23 - 50.87)	0.02 (0.00 - 1019.19)
Reoperation	0.85 (0.36 - 2.00)	0.98 (0.87 - 1.10)	0.90 (0.85 - 0.95)	0.68 (0.65 - 0.71)	1.11 (0.94 - 1.31)	1.18 (0.60 - 2.33)	1.01 (0.60 - 1.71)
**Composite Primary Adverse Events**	0.82 (0.50 - 1.35)	0.73 (0.68 - 0.78)	0.77 (0.74 - 0.80)	0.65 (0.62 - 0.67)	1.06 (0.92 - 1.23)	1.29 (0.76 - 2.18)	1.08 (0.73 - 1.61)
Superficial Incisional SSI	0.69 (0.24 - 2.00)	1.38 (1.20 - 1.59)	1.00 (0.94 - 1.06)	0.66 (0.63 - 0.69)	1.37 (1.17 - 1.59)	0.66 (0.31 - 1.40)	1.23 (0.74 - 2.05)
Deep Incisional SSI	1.49 (0.31 - 7.24)	1.20 (0.99 - 1.47)	0.82 (0.74 - 0.90)	0.56 (0.51 - 0.62)	1.37 (0.99 - 1.89)	1.48 (0.49 - 4.47)	1.22 (0.59 - 2.51)
Organ/Space SSI	1.31 (0.60 - 2.87)	0.85 (0.76 - 0.94)	0.71 (0.67 - 0.75)	0.69 (0.65 - 0.72)	1.25 (1.06 - 1.47)	1.03 (0.48 - 2.20)	0.81 (0.40 - 1.64)
Wound Disruption	0.71 (0.21 - 2.38)	0.97 (0.81 - 1.15)	0.85 (0.77 - 0.93)	0.53 (0.48 - 0.58)	0.98 (0.69 - 1.40)	0.13 (0.00 - 29.58)	***
Pneumonia	1.55 (0.80 - 3.03)	0.87 (0.79 - 0.96)	0.79 (0.74 - 0.84)	0.64 (0.61 - 0.68)	1.13 (0.91 - 1.42)	1.27 (0.57 - 2.86)	0.95 (0.49 - 1.85)
Urinary Tract Infection	3.58 (1.21 - 10.60)	0.92 (0.80 - 1.05)	0.79 (0.73 - 0.84)	0.75 (0.71 - 0.80)	1.17 (0.96 - 1.43)	1.45 (0.57 - 3.67)	0.32 (0.08 - 1.30)

Sepsis	1.21 (0.61 - 2.39)	1.15 (1.05 - 1.26)	0.74 (0.70 - 0.78)	0.58 (0.56 - 0.61)	1.21 (1.02 - 1.43)	1.11 (0.58 - 2.14)	1.36 (0.88 - 2.10)
Septic Shock	1.82 (1.01 - 3.28)	0.67 (0.62 - 0.74)	0.71 (0.67 - 0.75)	0.53 (0.49 - 0.56)	1.42 (1.09 - 1.84)	2.65 (1.21 - 5.82)	0.51 (0.21 - 1.24)
Unplanned Intubation	1.43 (0.69 - 2.96)	0.75 (0.67 - 0.83)	0.77 (0.72 - 0.82)	0.59 (0.55 - 0.63)	0.94 (0.71 - 1.24)	1.54 (0.65 - 3.65)	1.52 (0.90 - 2.59)
On Ventilator greater than 48 Hours	0.63 (0.35 - 1.12)	0.72 (0.66 - 0.79)	0.71 (0.67 - 0.75)	0.57 (0.54 - 0.61)	1.36 (1.08 - 1.72)	0.96 (0.40 - 2.27)	0.98 (0.48 - 1.99)
Pulmonary Embolism	2.33 (0.33 - 16.42)	0.76 (0.59 - 0.98)	0.78 (0.69 - 0.89)	0.65 (0.59 - 0.72)	1.08 (0.75 - 1.56)	0.62 (0.11 - 3.64)	1.66 (0.61 - 4.57)
DVT Requiring Therapy	0.99 (0.39 - 2.53)	0.77 (0.67 - 0.89)	0.61 (0.56 - 0.66)	0.62 (0.57 - 0.67)	0.90 (0.68 - 1.21)	1.15 (0.26 - 5.16)	0.44 (0.06 - 3.27)
Acute Renal Failure	0.76 (0.29 - 1.98)	0.83 (0.71 - 0.97)	0.79 (0.72 - 0.87)	0.46 (0.41 - 0.51)	1.66 (1.07 - 2.60)	0.33 (0.03 - 3.37)	1.32 (0.28 - 6.30)
Transfusions	1.43 (0.89 - 2.28)	0.70 (0.65 - 0.74)	0.50 (0.48 - 0.52)	0.53 (0.51 - 0.54)	1.08 (0.97 - 1.20)	1.92 (1.28 - 2.89)	0.81 (0.57 - 1.14)
Unplanned Readmission	0.71 (0.30 - 1.67)	1.16 (1.05 - 1.29)	0.96 (0.91 - 1.00)	0.74 (0.71 - 0.76)	1.10 (0.97 - 1.24)	0.72 (0.41 - 1.24)	1.52 (1.07 - 2.15)
Prolonged length of stay	1.02 (0.65 - 1.58)	0.92 (0.87 - 0.98)	0.43 (0.42 - 0.44)	0.52 (0.51 - 0.53)	1.16 (1.08 - 1.25)	2.64 (1.97 - 3.53)	0.63 (0.48 - 0.81)
**Composite Secondary Adverse Events**	1.64 (0.93 - 2.89)	0.87 (0.81 - 0.94)	0.43 (0.41 - 0.44)	0.56 (0.55 - 0.57)	1.13 (1.07 - 1.20)	2.10 (1.61 - 2.72)	0.81 (0.66 - 0.99)

***Spline analysis failed as no patients with serum albumin concentration > 6.4 g/dL experienced wound disruption

OR – odds ratio, CI – confidence interval; CPR – cardiopulmonary resuscitation; CVA – cerebrovascular accident; SSI – surgical site infection; DVT – deep venous thrombosis.
